# Training programs to improve identification of sick newborns and care-seeking from a health facility in low- and middle-income countries: a scoping review

**DOI:** 10.1186/s12884-021-04240-3

**Published:** 2021-12-14

**Authors:** Alastair Fung, Elisabeth Hamilton, Elsabé Du Plessis, Nicole Askin, Lisa Avery, Maryanne Crockett

**Affiliations:** 1 Hospital for Sick Children, Division of Paediatric Medicine, University of Toronto, 555 University Ave., Rm 10402, Black Wing, Toronto, Ontario M5G 1X8 Canada; 2 Institute for Global Public Health, Rady Faculty of Health Sciences, University of Manitoba, R070 Med Rehab Bldg, 771 McDermot Avenue, Winnipeg, Manitoba R3E 0T6 Canada; 3 Neil John Maclean Health Sciences Library, Rady Faculty of Health Sciences, University of Manitoba, 727 McDermot Avenue, Winnipeg, Manitoba R3E 0T6 Canada; 4 Institute for Global Public Health, Department Of Obstetrics, Gynecology and Reproductive Sciences, Rady Faculty of Health Sciences, University of Manitoba, R070 Med Rehab Bldg, 771 McDermot Avenue, Winnipeg, Manitoba R3E 0T6 Canada; 5 Institute for Global Public Health, Department of Pediatrics and Child Health, Medical Microbiology and Infectious Diseases, Community Health Sciences, Rady Faculty of Health Sciences, University of Manitoba, R070 Med Rehab Bldg, 771 McDermot Avenue, Winnipeg, Manitoba R3E 0T6 Canada

**Keywords:** Newborn care, Newborn, Neonatal, Training, Care-seeking, Low- and middle-income countries, Human resources for health

## Abstract

**Background:**

Most neonatal deaths occur in low- and middle-income countries (LMICs). Limited recommendations are available on the optimal personnel and training required to improve identification of sick newborns and care-seeking from a health facility. We conducted a scoping review to map the key components required to design an effective newborn care training program for community-based health workers (CBHWs) to improve identification of sick newborns and care-seeking from a health facility in LMICs.

**Methods:**

We searched multiple databases from 1990 to March 2020. Employing iterative scoping review methodology, we narrowed our inclusion criteria as we became more familiar with the evidence base. We initially included any manuscripts that captured the concepts of “postnatal care providers,” “neonates” and “LMICs.” We subsequently included articles that investigated the effectiveness of newborn care provision by CBHWs, defined as non-professional paid or volunteer health workers based in communities, and their training programs in improving identification of newborns with serious illness and care-seeking from a health facility in LMICs.

**Results:**

Of 11,647 articles identified, 635 met initial inclusion criteria. Among these initial results, 35 studies met the revised inclusion criteria. Studies represented 11 different types of newborn care providers in 11 countries. The most commonly studied providers were community health workers. Key outcomes to be measured when designing a training program and intervention to increase appropriate assessment of sick newborns at a health facility include high newborn care provider and caregiver knowledge of newborn danger signs, accurate provider and caregiver identification of sick newborns and appropriate care-seeking from a health facility either through caregiver referral compliance or caregivers seeking care themselves. Key components to consider to achieve these outcomes include facilitators: sufficient duration of training, refresher training, supervision and community engagement; barriers: context-specific perceptions of newborn illness and gender roles that may deter care-seeking; and components with unclear benefit: qualifications prior to training and incentives and remuneration.

**Conclusion:**

Evidence regarding key components and outcomes of newborn care training programs to improve CBHW identification of sick newborns and care-seeking can inform future newborn care training design in LMICs. These training components must be adapted to country-specific contexts.

**Supplementary Information:**

The online version contains supplementary material available at 10.1186/s12884-021-04240-3.

## Background

While neonatal and child mortality decreased substantially worldwide from 1990 to 2019, the burden of under-five mortality remains significant in low- and middle-income countries (LMICs) [1]. Indeed, LMICs did not achieve the Millennium Development Goal 4 (MDG4), which aimed to reduce under-five mortality by two-thirds between 1990 and 2015 [2]. The MDG4 translated to a 4% annual rate of decrease in under-five mortality during this period [2]. From 1990 to 2000, the annual global rate of decrease in under-five mortality only reached an average of 2% per year [3].

Moreover, survival rates differ among age groups of children younger than five years with a particularly high burden in the neonatal period (first 28 days of life). In 2019, 47% (2.4 million) of under-5 deaths occurred among neonates [1]. Mortality for neonates has also decreased at a much slower rate than those recorded for post-neonatal children [1]. Most neonatal deaths occur in LMICs with sub-Saharan Africa accounting for 42% of neonatal mortality in 2019, and Central and Southern Asia accounting for 37% [1]. Thus, reducing under-five mortality requires heightened attention to improving neonatal care in LMICs.

Over the past two decades, considerable progress has been made in improving the proportion of births attended by skilled health personnel (i.e., doctors, nurses or midwives) and occurring in a health facility in LMICs. From the period 2000–2006 to 2014–2020, the proportion of births occurring at a health facility in West and Central Africa, Eastern and Southern Africa, and South Asia increased from 38 to 55%, 37 to 66%, and 31 to 74%, respectively [4]. While coverage of facility-based deliveries has improved in LMICs, community-based follow-up of infants, particularly high-risk small and sick newborns, remains important to reduce neonatal morbidity and mortality [5].

Of all neonatal deaths globally, about two thirds occur after the first 24 h of life and almost three-quarters of neonatal deaths occur during the first week of life [6]. Moreover, about one half of neonatal deaths due to sepsis in LMICs occur after the first week of life [7]. Studies in various LMIC settings have shown that caregiver delay in recognizing sick newborns and in deciding to seek care are major contributors to neonatal mortality [8, 9]. A systematic review of care-seeking for neonatal illness in LMICs reported that a median of 59% of caregivers seek any care for their neonate once they recognize their neonate is ill or suspected to be ill [10]. Thus, improving community-based recognition of sick newborns and care-seeking from a health facility is critical.

The World Health Organization (WHO) 2013 recommendations on postnatal care of the mother and newborn [11] and the WHO recommendations on newborn health updated in 2017 [18], provide a summary of the best practices and supporting evidence for the timing, number and place of postnatal contacts as well as the content of newborn care during the first six weeks after birth. These guidelines are targeted at first level health workers or community level workers in resource-limited settings [11]. However, there is no specific mention of the optimal personnel and training required for effective newborn care provision. Regarding the place of newborn care, one systematic review concluded that community health worker (CHW) home visits resulted in a significant (18%) reduction in all-cause neonatal mortality compared to routine care [12]. A recent overview of systematic reviews provides evidence for interventions to prevent neonatal mortality but without specifically reviewing different care providers of these interventions or their training [13].

In 2015, the United Nations created the Sustainable Development Goals (SDGs) as the successor to the MDGs. Centred on reducing child mortality, SDG3.2 aims to decrease neonatal mortality to fewer than 12 deaths per 1000 livebirths and end preventable deaths of newborns and children under five years of age [14]. More than 60 countries are projected to miss this neonatal mortality target by 2030 and will need to accelerate progress to reach the target on time [1]. In light of these global goals and current trends, it is important to identify and map the optimal personnel and training required to deliver the most appropriate newborn care in LMICs. This scoping review aims to determine what is known from available literature about the relative effectiveness of community-based health worker (CBHW) newborn care providers and their training programs in LMICs in addition to mapping the key components necessary to design an effective CBHW newborn care training program and intervention to improve identification of sick newborns and care-seeking from a health facility in LMICs.

## Methods

### Search strategy and selection criteria

This study followed the PRISMA-ScR checklist (see Additional file 1) and the Joanna Briggs Institute (JBI) methodology for scoping reviews [15]. We employed an iterative approach to study selection whereby we narrowed our inclusion criteria as we became more familiar with the evidence base. Initial inclusion criteria were studies that: 1) included care providers for neonates defined as infants ≤28 days; 2) compared care providers or compared specific care providers to routine or no care for neonates; and 3) were conducted in LMICs. Care providers were defined as supplemental personnel outside of the newborn’s family offering newborn care. Newborn care practices were defined as any action taken by care providers that served to support the essential biological and psychosocial needs of the newborn following delivery to the first 28 days of life. Low- and middle-income economies were defined according to the World Bank classification [14]. No restrictions were placed on language.

A medical research librarian constructed the search strategy. An initial limited search of two databases (Medline and CINAHL) with search terms reflecting the concepts of “low/middle-income countries”, “postnatal care” and “provider type” was performed. We conducted an analysis of the results from the preliminary search to inform the development of the full search strategy, which was conducted across Medline (Ovid), CINAHL (EBSCO), Global Health (Ovid), the Cochrane Library collection of databases (Wiley), and DARE (Database of Abstracts of Reviews of Effects) from 1990 to March 31, 2020. We also searched the grey literature including all registered clinical trials on clinicaltrials.gov. See Additional file 2 for the full electronic search strategy in Medline (Ovid).

Among the articles included in the initial search, major areas of study within newborn care included newborn resuscitation, breastfeeding promotion, kangaroo mother care, umbilical cord care, prevention of mother-to-child transmission of HIV (PMTCT), identification of sick newborns and care-seeking from a health facility. After reviewing these various areas, we decided by consensus to narrow our inclusion criteria to focus specifically on studies of CBHW newborn care provision and training programs to improve identification of sick newborns and care-seeking in community-based settings outside of the hospital and primary health care facilities. Thus, the revised inclusion criteria were studies that: 1) investigated the effectiveness of CBHW newborn care providers and their training programs to improve identification of newborns with serious illness and/or care-seeking from a health facility, and 2) occurred in community-based settings in urban or rural areas of LMICs.

We defined CBHWs as health workers who meet the following criteria: 1) have some training in carrying out functions related to biomedical health care delivery, 2) have no formal professional or paraprofessional certificate, 3) provide care in community settings, that is, at patients’ homes or based at peripheral health posts not staffed by physicians or nurses, and not at primary health care facilities, and 4) are either paid or volunteer [16, 17]. ‘Newborns with serious illness’ was defined as newborns with ‘danger signs’ which included the WHO newborn danger signs [18], WHO signs of possible serious bacterial infection [19] and variations of these lists of signs.

We excluded studies conducted in neonatal intensive care units or tertiary care centres, studies conducted in high-income countries, review articles, conference abstracts, study protocols, case reports, commentaries and editorials.

### Data analysis

The total number of studies was divided among three reviewers (AF, EH and EdP) and two of the three reviewers independently performed the eligibility assessment for each study in a standardized manner using predefined inclusion and exclusion criteria, first in abstract form followed by full-text format. Disparities between reviewers were resolved by discussion and where there was still lack of agreement, a fourth reviewer (MC) was consulted.

Two reviewers (AF and EH) extracted the data from all included studies onto a data extraction form. Information collected included first author and year of publication, country and study setting, study design, characteristics of the neonatal subjects, characteristics of the newborn care providers, duration and content of training if available, the content and coverage of the intervention and the outcomes as they pertained specifically to the identification of newborns with serious illness and care-seeking from a health facility.

We did not perform a formal assessment of methodological quality as this is not a typical feature of scoping reviews [15].

## Results

**Fig. 1:**
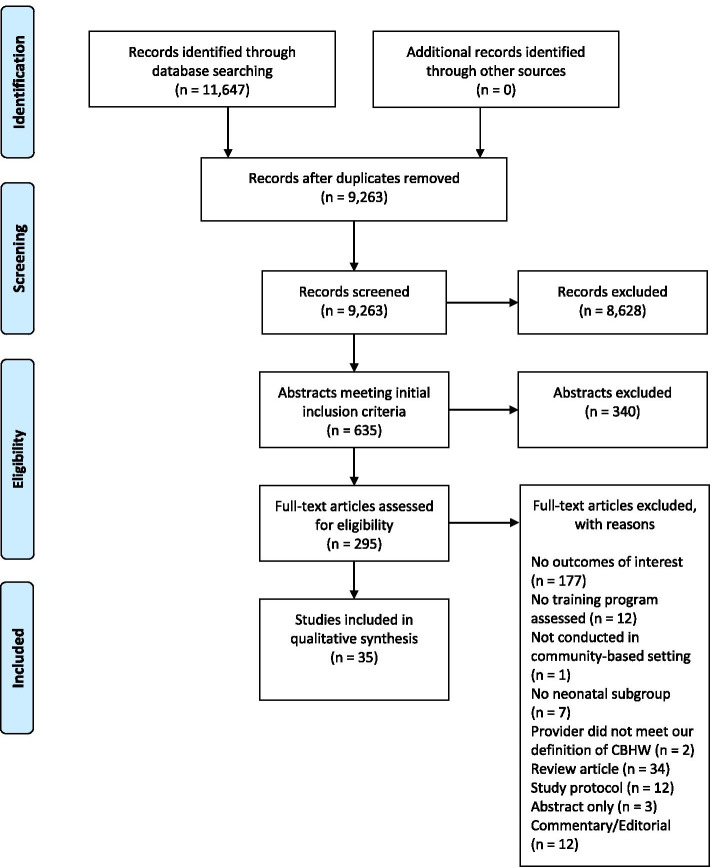
Flow diagram for study selection

The flow diagram of study selection is shown in Fig. 1.

The search strategy identified 11,647 articles. After adjusting for duplicates, 9263 studies remained. Of these, 635 studies met the initial inclusion criteria. Within these results, 35 articles [20–54] detailing 28 studies met the revised inclusion criteria. The characteristics of the 35 included articles are summarized in Table 1 and in an expanded Table in Additional file 3.

**Table 1:** Characteristics of studies on newborn care providers in low-and middle-income countries

			Training				Knowledge of newborn danger signs		Identification of newborns with serious illness		Care seeking	Key results
Authors & Year	Country	Newborn care provider	Intervention or training	Duration	Content	Monitoring or refresher training	Provider	Caregiver	Provider	Caregiver		
Ansah et al. 2014 [20]	Ghana	Community based-surveillance volunteer (CBSV)	Training program for home visits by CBSVs	9 days	3 days on behaviour change communication, counseling skills, promotion of essential newborn care practices; 4 days on assessment and referrals; 2 days of refresher course and clinical practice sessions in major health facilities.	Yes	Not assessed	Not assessed	Assessed	Not assessed	Not assessed	CBSVs’ newborn assessments strongly agreed with district-based project supervisors (DiPS) with coefficients of agreement between 0.75–1.0. The sensitivities of CBSVs’ diagnosis for signs checked by observation were relatively low (57–59%) with just >40% detected by the DiPS missed by the CBSV. However, specificities were close to 100% for all danger signs. Referral decisions made by the CBSVs also achieved excellent agreement with the DiPS; Kappa = 0.87 (0.82, 0.92), with 80% sensitivity and 100% specificity.
Bang et al. 2005a [21]	India	Village health worker (VHW)	Health education, including newborn danger signs	6 months	Taking histories of pregnant women, observing labor, examining neonates, recording findings, case management of pneumonia in neonates, supporting mothers with home neonatal care and identifying neonatal morbidities.	Yes	Not assessed	Assessed	Not assessed	Assessed	Not assessed	Regarding caregiver knowledge of danger signals in a baby when the VHW should be called, 77.3% of mothers correctly responded. Regarding caregiver identification of newborns with serious illness, 87.2% of mother correctly called a VHW if a baby was sick.
Bang et al. 2005b [22]	India	VHW	VHW home visits	2 months	Taking a history, examining a mother and newborn, and recording data. Trained to give intramuscular vitamin K, diagnose and treat sepsis.	Yes	Not assessed	Not assessed	Assessed	Not assessed	Assessed	VHWs correctly diagnosed 492 (89.1%) of neonatal sepsis cases compared to a computer algorithm. Of the 492 patients diagnosed by the VHW to have presumed sepsis, parents agreed to and were able to hospitalize only 13 infants (2.6%), but agreed to homebased treatment for almost all infants (91.1%). Of note, in 31 cases (6.3%), parents refused both home and hospital care.
Bang et al. 2005c [23]	India	VHW	VHW home visits	2 months	Taking a history, examining a mother and newborn, and recording data. Trained to give intramuscular vitamin K, diagnose and treat sepsis.	Yes	Not assessed	Not assessed	Assessed	Assessed	Not assessed	Among LBW or preterm infants or both, VHWs correctly diagnosed sepsis 94% of the time and correctly treated sepsis 95% of the time.
Baqui et al. 2009a [24]	Bangladesh	Community health worker (CHW)	Performance of CHWs in assessing neonates using an Integrated Management of Childhood Illness (IMCI)-type algorithm, within the home care arm of the trial	6 weeks	Skills development for behaviour change communication, clinical assessment of neonates, treatment of newborns with injectable antibiotics and record-keeping; hands-on clinical training under supervision in a tertiary care hospital and in households.	Yes	Not assessed	Not assessed	Assessed	Not assessed	Not assessed	Compared to physician assessment, CHWs are able to correctly classify very severe disease in newborns with a sensitivity of 91%, specificity of 95%, and kappa value of 0.85 (*p* < 0.001). CHW recognition of newborn signs and symptoms showed a sensitivity of more than 60% and a specificity of 97–100%.
Baqui et al. 2009b [25]	Bangladesh	CHW	Analysis of data in home care arm (see Baqui et al. 2009a)	6 weeks	As above.	Yes	Not assessed	Not assessed	Assessed	Not assessed	Assessed	CHW home-based treatment of very severe disease in neonates was acceptable and resulted in a hazard ratio for death of 0.22 (95%CI 0.07–0.71) compared to those who received no treatment or were treated by untrained providers.
Bari et al. 2006 [26]	Bangladesh	CHW	Home visits by CHWs and educating families about danger signs in the postpartum period, examining newborns for danger signs, increasing referral compliance	1 month	Skills and knowledge on maternal and newborn care.	Yes	Not assessed	Not assessed	Not assessed	Not assessed	Assessed	Compliance with referral by the CHWs increased from 55.7% during the first three-month period of implementation to 80.1% during the third three-month period of implementation and was thereafter maintained at 75–80%. There was a highly significant (*p* < 0.00001) increase in the proportion of families that sought care from qualified providers for sick newborns in the intervention arm and a non-significant increase in the comparison arm.
Broughton et al. 2016 [27]	Ecuador	Traditional birth attendant (TBA)	Provincial-level network to coordinate maternal newborn health services and strengthened linkages between levels of care	4 days	TBAs and CHWs were trained to identify maternal and newborn danger signs and risk factors and to refer them to health centers.	Yes	Not assessed	Assessed	Assessed	Not assessed	Not assessed	More mothers could identify newborn danger signs at endline (83%) compared to baseline (30%). Providers were able to identify two or more newborn danger signs 86% (n = 311) in first year of project vs. 98% (n = 49) in last year of project.
Darmstadt et al. 2011 [28]	Bangladesh	CHW	Home visits by CHWs to assess signs and symptoms of neonatal illness	36 days	Pregnancy surveillance, counseling and negotiation skills, essential newborn care, neonatal illness surveillance and management of illness based on a clinical algorithm adapted from IMCI.	Yes	Not assessed	Not assessed	Assessed	Not assessed	Not assessed	Compared to physician assessment, use of a 6-sign algorithm by CHWs had a sensitivity of 81.3% and specificity of 96.0% for identifying infant referral need and a sensitivity of 58.0% and specificity of 93.2% for screening infant mortality.
Darmstadt et al. 2010 [29]	Bangladesh	CHW	Preventive and curative maternal-neonatal healthcare package	36 days	As above.	Yes	Not assessed	Assessed	Not assessed	Not assessed	Assessed	Improvements in unprompted caregiver knowledge of maternal and neonatal danger signs were significantly larger in the intervention arm compared to the comparison arm. Among neonates who had ≥1 of the 10 selected complication signs, care seeking from a qualified provider increased significantly more in the intervention arm (from 31 to 56%) than in the comparison arm (from 27 to 35%).
Darmstadt et al. 2009 [30]	Bangladesh	CHW	Home visits by CHWs to assess signs and symptoms of neonatal illness	36 days	As above.	Yes	Not assessed	Not assessed	Assessed	Not assessed	Not assessed	Compared to physician assessment, CHWs classified very severe disease in infants with a sensitivity of 73%, specificity of 98%, PPV of 57% and NPV of 99%.
Das et al. 2014 [31]	India	Accredited Social Health Activists (ASHA)	ASHA home visits	5 days	Orientation on state government Home-based Newborn Care (HBNC) recording formats, and an Integrated Management of Newborn and Childhood Illness (IMNCI) skills review.	No	Not assessed	Not assessed	Assessed	Not assessed	Not assessed	Compared to trained investigators, ASHA agreement on the need to further assess infants was intermediate (kappa = 0.48, *P* < 0.001) and overall ASHA-investigator agreement on diagnosis was poor (kappa = 0.23, *P* = 0.01).
Findley et al. 2013a [32]	Nigeria	Control vs. Community volunteer (CV) vs. CHW and CV	High intensity (CV + CHW) compared to low intensity (CV) intervention	2 weeks	Community based using train-the-trainers model.	No	Not assessed	Assessed	Not assessed	Not assessed	Assessed	At follow-up, most women knew at least one of the newborn danger signs, with the most commonly known danger sign being high fever, known by 83–84% of women, regardless of the level of intervention they had experienced (F = 0.182, *P* = 0.825). However, at follow-up, for almost all danger signs, women living in the intervention communities were more aware than those in the control communities.
Findley et al. 2013b [33]	Nigeria	Control vs. CHW & CV	Health promotion through system-wide changes in health planning and implementation	Not specified	Community based using train-the-trainers model.	No	Not assessed	Assessed	Not assessed	Not assessed	Not assessed	At the midterm follow-up, most women knew at least one of the newborn danger signs, with the most commonly known danger sign being high fever, known by 82.7% in the control and 84.2% in the intervention communities. Many women knew other critical danger signs that indicated the need for the baby to be seen by a health worker. In the intervention areas, 31.0% knew to worry about diarrhea, dehydration, and sunken fontanel and about fitting or convulsions, significantly more than in the control areas. Women in the intervention areas were also more likely to know about breathing problems and not being able to suckle or refusing to feed.
Gebremedhin et al. 2019 [49]	Ethiopia	Health extension worker (HEW) and nurse	Use of HEWs at community levels to strengthen linkages between health centers and health posts	Not specified	Community-based newborn care.	No	Assessed	Assessed	Not assessed	Not assessed	Assessed	Provider knowledge of newborn related indicators ranged from poor to good. 88.8, 75.4 and 70.1% of mothers knew fever, poor sucking or ability to suck and fast breathing were danger signs.
Goel et al. 2019 [50]	India	ASHA	National government strategy of HBNC implemented by ASHAs as a quality of care improvement intervention package	2 days	Refreshing of technical skills, communication skills and counseling techniques; early initiation of breastfeeding, thermal care, hand washing, restrictive handling, cord care, recognition of danger signs.	No	Assessed	Not assessed	Not assessed	Assessed	Assessed	Knowledge score went from 5.1 to 7.1 (max score 9), with 39.2% relative change from baseline. There was an improvement in the intervention area for a mother recognizing illness in a neonate in the first month (mean 15.3 (SE 8.8–25.3) in standard area vs. 31.9 (SE 22.1–43.6) in the intervention area *p* = 0.019), and mother contacting an ASHA for neonatal illness (mean 2.8 (SE 0.8–9.6) in the standard area vs. mean 23.2 (SE 14.8–34.4) in the intervention area (*p* < 0.001).
Gupta et al. 2017 [34]	India	Auxiliary nurse midwife	Microteaching technique for enhancing the postnatal care skills of health workers	17 months	Training content was developed from the gaps identified while observing health workers delivering postnatal care in the videos.	Yes	Not assessed	Not assessed	Assessed	Not assessed	Not assessed	Out of a maximum score of 2, newborn examination of the eye (0.07), limping of legs (0.07), feeding (0.14), abdominal examination (0.0), and chest indrawing (0.0) were poor (average score < 0.30) at the baseline, but showed significant (*p* < 0.001) improvement ranging from 1.50 to 1.75 at the end of the final round. Average neonatal examination skill score improved from 0.52 (Round 1) to 1.29 (Round 2) to 1.74 (Round 3). The overall skill assessment score significantly improved with each round of microteaching from 0.64 in the first round to 1.76 at the end of the third round.
Hodgins et al. 2010 [35]	Nepal	Female community health volunteer (FCHV)	Antenatal health education package	Not specified	District public health system supervisors and supervisors provided the bulk of the training and supervision.	No	Not assessed	Not assessed	Not assessed	Not assessed	Assessed	The percentage of respondents who sought care following recognition of danger signs increased for newborns: Baseline 51.0 (n = 310), Endline 59.4 (n = 414), OR: 1.42 (1.08–1.88). There was a decrease of about one half day in the time lag between illness onset and care seeking.
Horwood et al. 2017 [36]	South Africa	CHW supervisor	Quality improvement training for CHWs and supervisors	2 weeks	Community-based care of women and infants information on HIV and prevention of mother-to-child transmission (PMTCT), identification of signs of illness in newborn infants and children.	Yes	Not assessed	Assessed	Not assessed	Not assessed	Assessed	Mothers in the intervention group were more likely to have received a visit during pregnancy (75.7% vs. 29%, *p* < 0.0001) and postnatal period (72.6% vs. 30.3%, *p* < 0.0001). Mothers in the intervention group had higher maternal and child health knowledge scores (49 vs 43%, p = 0.007) and reported higher exclusive breastfeeding rates to 6 weeks (76.7 vs 65.1%, *p* = 0.02).
Khanal et al. 2011 [37]	Nepal	Facility-based community health worker (FB-CHW) & female community health volunteer (FCHV)	FCHV and FB-CHW use of MINI algorithm and subsequent administration of antibiotics	4 months	Assessment and management of neonatal infections and early newborn care (ENC) messages.	Yes	Not assessed	Not assessed	Assessed	Not assessed	Not assessed	FCHV assessments matched the more highly trained facility-based CHWs in over 90% of cases. Treatment was initiated in 90% of sepsis cases.
Kumar et al. 2008 [38]	India	CHW	Intervention comparing package of preventive essential newborn care with intervention group receiving essential newborn care plus liquid crystal sticker that indicates hypothermia by changing colour	7 days	Combination of classroom-based and apprenticeship-based field training on knowledge, attitudes, and practices related to essential newborn care within the community, behaviour change management, and trust-building.	No	Not assessed	Not assessed	Not assessed	Not assessed	Assessed	Improvements in birth preparedness, hygienic delivery, skin-to-skin care, umbilical cord care and breastfeeding were seen in intervention arms. Compared with controls, neonatal mortality rate was reduced by 54% in the essential newborn-care intervention (*p* < 0.0001) and by 52% in the essential newborn care plus ThermoSpot arm (*p* < 0·0001). There was little change in care-seeking.
Limaye et al. 2020 [51]	Bangladesh	Field worker (FW)	Educational digital health intervention providing netbook computers to FWs	2 days	eToolkits intended for use as a counseling tool and eLearning courses to supplement in-person trainings. Course included maternal and newborn health (MNH), family planning, nutrition, integrated messaging and interpersonal communication.	Yes	Assessed	Not assessed	Not assessed	Not assessed	Not assessed	Difference in mean scores in relation to newborn danger signs was significant from pre to post. Scores increased from 2.01 to 3.27 (range of 0–4) for a 1.25 mean score difference, significant at *p* < 0.001. Scores on how to care for pre-term infants increased from 0.56 to 1.49 (range 0–2) for a 0.93 difference, significant at *p* < 0.001.
Manu et al. 2016 [39]	Ghana	CBSV	Home visits in the first week of life; newborn care seeking promotion by CBSVs; dialogue and problem solving with families around barriers to seeking care	9 days	Interactive discussions, group exercises and practical newborn assessment video exercises.	Yes	Not assessed	Not assessed	Assessed	Not assessed	Assessed	Almost 70% of recently delivered women received CBSV assessments. Compliance with referrals was very high, especially in the poorest quintile. Independent care seeking for severe newborn illness increased from 55.4% in control to 77.3% in intervention zones. This was a near doubling among the poorest quintile.
Mascarenas et al. 2015 [40]	Kenya	CHW	Training program for CHWs to detect clinical signs that predict severe illness in children under two months of age	5 days plus 2 days on neonatal danger signs	Signs and symptoms of neonatal illness on a routine home visit during the first week of life, and determining need for referral to a health facility, methods of malaria control, childhood respiratory tract infections and diarrheal disease, domestic and personal hygiene, child nutrition, reproductive health, HIV/AIDS, and communication skills.	No	Not assessed	Not assessed	Not assessed	Not assessed	Assessed	35% of families who didn’t receive a CHW visit reported taking their infant to a healthcare facility, compared with 21% of families who did receive a CHW-visit (*p* < 0.01). Rates of overnight hospitalization were 6% for not-visited infants and 1% for visited infants (*p* < 0.01).
McConnell et al. 2016 [41]	Kenya	CHW	Phone calls using checklist by CHWs to newly delivered mothers	4 days	Conducting screenings using the checklist and counseling mothers and caregivers on essential postnatal health education.	No	Not assessed	Assessed	Assessed	Not assessed	Assessed	85% of women in the home visit arm were able to name 3+ maternal danger signs and 3+ infant danger signs. 81 and 78% in the phone call arm were able to name 3+ maternal danger signs and 3+ infant danger signs, respectively. 79 and 67% of women in the standard of care arm were able to name 3+ maternal and infant danger signs. Facility-based care seeking for infants was high in all arms at 94–96%. However, the differences in timing of infant-related care seeking was statistically different between arms. Women in the phone call and home visit sought care for their baby 2.0 (*p* = 0.014) and 1.8 days (*p* = 0.034) earlier than the standard of care.
Mozumdar et al. 2018 [52]	India	Peer health educator	Home-based maternal and newborn care (HBMNC) messaging by self-help groups	1 day	Key HBMNC topics, conducting discussions on health topics and the different health services that are available at public health facilities.	Yes	Not assessed	Assessed	Not assessed	Not assessed	Not assessed	Women in the experimental area showed significant knowledge increase in importance of ANC, danger signs for newborn child, skin to skin care, KMC and delayed bathing. Endline results also showed significant increase in knowledge for cord care, delayed bathing and KMC. In the experimental arm, knowledge of danger signs for a newborn child all had significant increases, except for chest indrawing. For the comparison group, some danger signs showed a loss of knowledge between baseline and endline.
Nalwadda et al. 2013a [42]	Uganda	CHW	CHW visits	Not specified	Counseling for caretakers with referred newborns; newborn danger signs.	No	Not assessed	Not assessed	Not assessed	Not assessed	Assessed	700 newborns were referred by CHWs and successfully traced. Of these, 373 (53%) were referred for immunization and postnatal-care, and 327 (47%) were referred for a danger-sign. 439 (63%) complied, and of the 327 newborns with a danger sign, 243 (74%) caretakers complied with the referrals.
Nalwadda et al. 2013b [43]	Uganda	CHW	CHW home visit	5 days	CHWs’ roles during pregnancy and after delivery including health education, screening for danger signs and counseling for referral.	Yes	Assessed	Not assessed	Assessed	Not assessed	Not assessed	68% of the CHWs attained the pass mark for knowledge scores. 74% mentioned the required five newborn danger signs; chest in-drawing and grunting were never mentioned as newborn danger signs. 63% attained the pass mark for both skills and communication. 98% correctly identified the four case-vignettes as a sick or not sick newborn. Preterm birth was the least identified danger sign from the case-vignettes.
Namazzi et al. 2017 [44]	Uganda	CHW	Community mobilization & sensitization using CHWs and radio talk shows	5 days	Home visits and community dialogues on maternal and newborn issues.	Yes	Assessed	Not assessed	Not assessed	Not assessed	Not assessed	CHWs’ knowledge of maternal and newborn health improved from 41.3 to 77.4% after training, and to 79.9% one year after training. Main predictors of knowledge were age and level of education.
Ndaba et al. 2019 [53]	South Africa	CHW	Lectures, demonstrations and individual and group exercises; simulated home visits; training manual based on UNICEF and WHO materials focused on home visits, support and care, child feeding practices and postnatal care at a community level	3 days	Skills and knowledge to assist pregnant mothers and their babies pre and post-delivery at their homes in the first month of life; to conduct home visits during pregnancy and after delivery; and to enhance a good working relationship with the Community Advisory Group and Partner Defined Quality structures.	Yes	Assessed	Not assessed	Not assessed	Not assessed	Not assessed	CHWs’ knowledge of MNH improved from 63 to 72% (clinic A), 38 to 66% (clinic B), 48 to 50% (clinic C). The test focused on home care and support, child feeding and clinical care and support for MNH.
Nsibande et al. 2013 [45]	South Africa	CHW	CHW home visits	2 weeks	Role plays that were video recorded and content used for teaching and supervision purposes.	No	Not assessed	Not assessed	Not assessed	Not assessed	Assessed	95% of mothers completed the referral advised by CHWs, with 22% occurring during the first two weeks of life. For completed referrals, 51% of mothers could recognize danger signs that required care seeking (*p* = 0.01). The mothers who did not complete referral were unable to recognize infant danger signs.
Rahman et al. 2019 [54]	Bangladesh	CHW	CHW meetings with women and courtyard meetings with husbands and community members.	Not specified	Build capacities of women (promote birth preparedness and complication readiness, increase awareness of rights and MNH needs and increase health providers’ capacity to counsel women) and engage families and communities (male involvement and community involvement).	No	Not assessed	Assessed	Not assessed	Not assessed	Assessed	In the intervention area, women who were aware of at least 3 danger signs for newborns increased from 63 to 83% while knowledge declined in the comparison area. For all knowledge categories related to danger signs, the intervention had significantly increased levels. Regarding newborn care from a skilled health professional, there was no notable improvement between baseline and endline in either comparison or intervention areas.
Soofi et al. 2017 [46]	Pakistan	Lady health worker (LHW)	LHWs linked with traditional birth attendants and encouraged to attend home births.	5 days’ training for LHW programme master trainers; LHWs received initial 3 days of training and monthly 1 day sessions	High-risk pregnancies, antenatal care (ANC) care and other standard pregnancy advice, neonatal danger signs and illness with focus on neonatal sepsis; TBAs received basic essential neonatal care training and linkages with LHWs; support groups received training in communication, and counseling skills and knowledge on birth asphyxia, low birthweight, and sepsis; health-care providers received training on essential neonatal care and management of birth asphyxia, low-birthweight babies, and neonatal sepsis, provision of inflatable bag and mask and oral amoxicillin for sepsis with management protocols.	Yes	Not assessed	Not assessed	Not assessed	Not assessed	Assessed	For care seeking for neonates with reported illness, there was no significant difference between control or intervention (93 vs 93%, *p* = 0.89) However, there was a significant difference between control and intervention groups for neonates with possible infections who were seen by a LHW (2 vs. 29% (*p* < 0.001)).
Tripathy et al. 2016 [47]	India	ASHA	ASHA-led women’s group meetings	11 days	Not specified.	Yes	Not assessed	Not assessed	Not assessed	Not assessed	Assessed	Improvements in care seeking for newborn health problems were seen in both arms. From baseline to evaluation, the intervention arm improved from 46 to 72% and the control arm moved from 60 to 77%.
Waiswa et al. 2015 [48]	Uganda	CHW	Home visit package with health facility strengthening	5 days	Goal-oriented ANC, managing maternal complications, infection prevention, managing normal labour and partograph use, neonatal resuscitation, care of the sick newborn, and extra care for the small baby using kangaroo mother care.	Yes	Not assessed	Not assessed	Not assessed	Not assessed	Assessed	Improvements were seen in essential newborn care practices between intervention and control arms. Almost half (49.6%) of the mothers in the intervention waited more than 24 h to bathe the baby, compared to 35.5% in the control arm (*p* < 0.001) and dry umbilical cord care was also significantly higher in intervention areas (63.9% vs. 53.1%, *p* < 0.001). Immediate and exclusive breastfeeding also saw improvements between intervention and control arms (72.6% vs. 66.0%; *p* = 0.016 and 81.8% vs. 75.9%, *p* = 0.042, respectively). There was no difference in care-seeking for newborn illness in either arm but it was already high at 95%.

*Abbreviations: ANC* Antenatal care, *HEW* Health extension worker, *ASHA* Accredited Social Health Activist, *IMNCI* Integrated management of newborn and childhood illness, *CBSV* Community-based surveillance volunteer, *IMCI* Integrated management of childhood illness, *CHW* Community health worker, LHV- Lady health volunteer, *FB-CHW* Facility-based community health worker, *MNH* Maternal and newborn health, *FCHV* Female community health volunteer, *PMTCT* Prevention of mother-to-child transmission, *FW* Field worker, *TBA* Traditional birth attendant, *HBMNC* home based maternal and newborn care, *VHW* Village health worker, *HBNC* Home based newborn care,

**Fig. 2:**
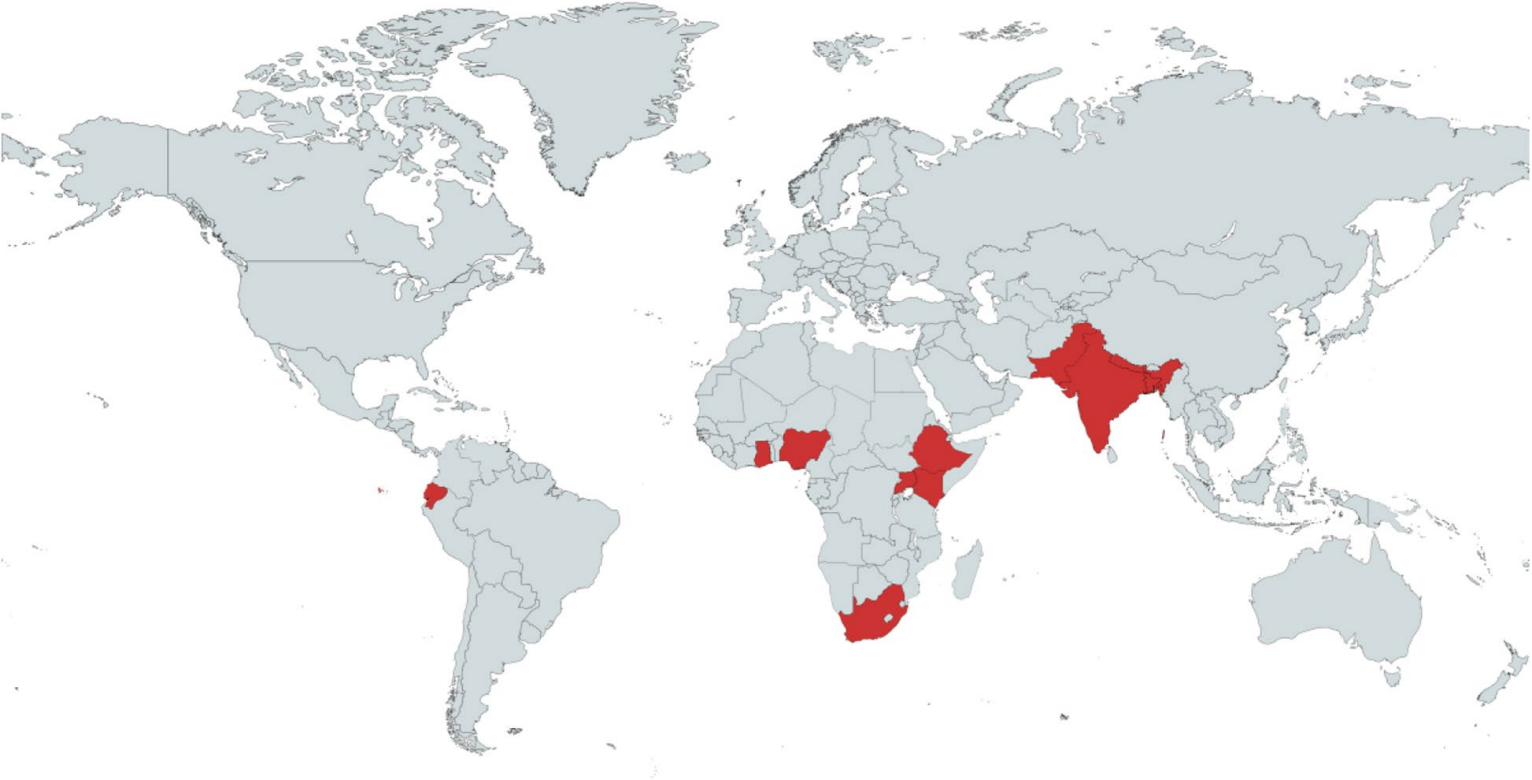
Countries represented in included articles

The included articles were published between 2005 and 2020. Studies represented 11 countries across three sub-continents (Fig. 2).

### Newborn care provider types

Eleven community-based newborn care provider types were identified. These included community health workers (CHWs), village health workers (VHWs), traditional birth attendants (TBAs), accredited social health activists (ASHAs), auxiliary nurse midwives (ANMs), female community health volunteers (FCHVs), community-based surveillance volunteers (CBSVs), community volunteers (CVs), lady health workers (LHWs), health extension workers (HEW) and peer educators. The most common provider type was the CHW.

### Platforms and timing of care delivery

Thirty-one studies used home visits as the platform of care delivery. One study investigated women’s group meetings [47]. Another study compared “high intensity” intervention, “low intensity” intervention and control groups, with “high intensity” consisting of CHW home visits and CV outreach and community engagement through group discussions and “low intensity” consisting of CV outreach and engagement activities only [32]. One study assessed CHW contacts with women either in their home or by mobile phone three days after delivery [41]. The number and timing of home visits varied widely across studies. The number of home visits ranged from one to eight in the first 28 days of life. The scheduling of home visits had even greater variation, ranging from two antenatal and three postnatal home visits in the first week after birth,to one intervention having home visits on days 1, 2, 3, 5, 7, 15, 21, 28 with high-risk newborns also having visits on days 4, 6, 9, 13 and 18. Overall, the most common number and schedule of visits was between three to five postnatal visits within the first 28 days, with visits on days 0, 3 and 7, one visit at the beginning of the second week of life and one visit towards the end of the 28-day period.

### Training content

The content of training varied but generally included history-taking, clinical assessment of mothers and newborns, identification of newborn danger signs, decision making for referral and facilitation of referral compliance, skills development for behaviour change communication, and data recording. Examples of newborn danger signs included: red umbilicus or cord with pus, newborn feeling hot or cold, failure to breastfeed, convulsions, rapid breathing, lack of body movement when stimulated, yellowing of the palms and soles, chest in-drawing and grunting [42]. Training and testing focused on visual assessments for newborn danger signs.

### Training methods

Approaches to training included didactic teaching, demonstrations, role-plays, simulation exercises, practicing physical examination on newborns, group discussions, effective communication and educational messages and train-the-trainer models. Case vignettes were used as teaching tools and photographs and audiovisual materials were used as references for various neonatal danger signs. One study employed a technique called “microteaching” which involved several rounds of video recording with ongoing provision of feedback to continually improve newborn care provider performance [34].

### Provider effectiveness in knowledge and identification of newborn danger signs

Six studies evaluated provider knowledge of newborn danger signs [43, 44, 49–51, 53]. The results of all six studies suggested that training programs improved provider knowledge of newborn danger signs. For example, Namazzi et al. reported that knowledge of newborn danger signs and essential home-based newborn care improved following the training, with pre/post mean test scores of 41.3% versus 77.4, and 79.9% one year later [44]. Thirteen studies included provider identification of newborns with serious illness as an outcome of interest [20, 22–25, 28, 30, 31, 34, 37, 39, 41, 43]. Overall, when comparing pre- and post-test results or baseline versus end line evaluations, CBHWs exhibited improved scores in identifying newborns with serious illness. Gupta et al. noted that there was significant improvement between baseline and the final round of assessments, with the skills assessment scores improving significantly after each round of microteaching. When CBHW assessments were compared with other more highly trained and experienced health cadres, the CBHW assessments obtained significant agreement with their counterparts [24, 25, 37]. Nalwadda et al. noted that, on average, seven of the 11 expected danger signs were identified with a median of 10, but pre-term birth was the least commonly identified danger sign [43].

### Key outcomes to be measured in an effective newborn care training program and intervention to improve identification of sick newborns and care-seeking from a health facility in LMICs

**Fig. 3:**
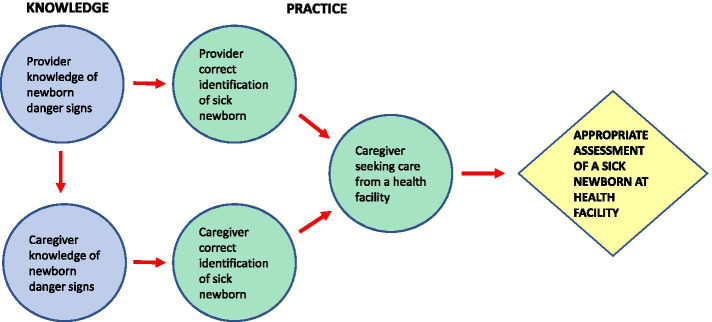
Key outcomes to be measured in a newborn care training program to increase appropriate assessment of a sick newborn at a health facility

The included studies demonstrated that training newborn care providers can improve identification of sick newborns and care-seeking from a health facility in LMICs. Increasing appropriate assessment of a sick newborn and referral to a health facility requires measurement of five key interrelated outcomes that cover both knowledge and practice of newborn care providers and caregivers: 1) provider knowledge of newborn danger signs, 2) caregiver knowledge of newborn danger signs (through education from provider), 3) correct identification of sick newborn by provider, 4) correct identification of sick newborn by caregiver and 5) appropriate care-seeking from a health facility (through compliance with provider referral or care-seeking from caregivers themselves) (Fig. 3). It is important for these key outcomes to be measured and evaluated at multiple time points during and after training and retraining.

### Key components to consider in the development of newborn care training programs to achieve key outcomes

The key components to consider in the design and implementation of a newborn care training program to achieve key outcomes are summarized in Table 2 as facilitators, barriers and components with unclear benefit.

**Table 2:** Key components to consider in the design and implementation of a newborn care training program to achieve key outcomes

Facilitators	Barriers	Components with unclear benefit
Sufficient duration of training Refresher training and retraining Supervision and on-the-job training Community engagement	Context-specific perceptions of newborn illness and gender roles that may deter care-seeking from a health facility	Qualifications and experience prior to initiating newborn care training Newborn care provider incentives or remuneration

### Facilitators

#### Sufficient duration of training

The median training duration was 39 days with a wide range among studies of four days to 17 months. One study showed intermediate ASHA to post-graduate female investigator agreement on the need for further assessment of infants (kappa 0.48, *P* = <0.001) [31]. Disagreement occurred for skin pustules, breastfeeding difficulty, incessant crying and infrequent newborn urination [31]. Compared to other studies, this study had a relatively short duration of training of five days. Three studies showed no difference in appropriate care-seeking by a caregiver between baseline and end line or between intervention and control groups [46–48]. Of note, these studies had relatively short training durations of three to 11 days.

#### Refresher training and retraining

Two studies assessed newborn care provider knowledge of newborn danger signs and both were conducted in Uganda [42, 44]. Nalwadda et al. evaluated CHWs 25 months after initial training and showed that almost all of the CHWs (56 out of 57, 98%) correctly identified all the prompted newborn danger signs in case-vignettes [42]. Namazzi et al. showed that CHW knowledge of newborn danger signs improved markedly from 20.8 to 85.5% following training [44]. However, after one year, the knowledge of three newborn danger signs dropped to 58.9% [44]. The duration of initial training in both studies was five days. Of note, in the study by Nalwadda et al., the CHWs met with their supervisors and were directly observed on a monthly basis following training to reinforce knowledge and skills. In another study, microteaching, that is, longitudinal video-recording of CHW patient encounters and providing regular feedback on strengths and weaknesses, led to improved newborn care provider ability to identify sick newborns over time [34]. The study by Namazzi et al. did not specify any refresher training.

Six studies showed that various newborn care providers including CBSVs, VHWs, CHWs and FCHVs were able to correctly classify sick newborns at home visits with high inter-rater agreement when compared to a gold standard (e.g., physicians, a computer algorithm, facility-based CHWs) [20, 22–24, 28, 30]. Common themes in the training programs of these studies included interactive simulated newborn assessment video exercises, behaviour change communication and counselling and repeated assessment and retraining until performance was deemed satisfactory.

#### Supervision and on-the-job training

Nine studies included supervision and on-the-job training as part of CBHW training programs [20, 22–26, 39, 43, 44]. In all of these studies, the CBHWs were provided with direct supportive supervision at regular intervals. These intervals ranged from two days per month during which a supervisor accompanied the CBHW to drop-in visits by the district supervisor once per month. Where there was supportive supervision, feedback was provided immediately at the time of observation, in addition to regularly scheduled meetings with larger groups of CBHWs where shared challenges could be discussed and solutions developed as a group. In most studies, the supervisors were within the community health network. In the study by Namazzi et al., the supervisors were “super CHWs” who conducted observed supervision during home visits and facilitated regular meetings with CHWs, supervisors and district health management teams [44]. In four studies [20, 22, 23, 39], supervisors also ensured that supplies needed for CBHW home visits were maintained while observing and participating in home visits. Seven of the nine studies that included supervision or on-the-job training evaluated provider identification of newborn danger signs [20, 22–24, 39, 43, 44]. Results from these studies suggested that CBHWs were able to accurately assess newborns for danger signs. By contrast, in the study by Das et al., which did not mention supervision or on-the-job training, ASHAs failed to assess many critical newborn danger signs and there was poor agreement between ASHA and trained postgraduate female investigator assessments of illness severity (kappa = 0.23, P = 0.01) [31]. Based on these results, Das et al. highlighted the importance of ongoing supportive supervision to ensure CBHWs’ long-term retention of illness assessment skills [31].

#### Community engagement

Eleven studies assessed caregiver knowledge of newborn danger signs and all showed significant improvements in this outcome in post-intervention groups compared to pre-intervention groups and in intervention groups compared to control groups [21, 27, 29, 32, 33, 36, 41, 49, 50, 52, 54]. Among these studies, integration of newborn care training and interventions into the community through community engagement was an important theme. In addition to providers teaching caregivers how to recognize newborn danger signs, one study included a weekly radio program providing information on danger signs, healthy behaviours and seeking professional care [27], one study utilized CV community outreach and engagement [32] and another study utilized mobile phone contacts [41]. Of note, CV community outreach and engagement and mobile phone CHW contacts individually performed similarly to home visits and led to significant improvement in caregiver knowledge of danger signs compared to the control [32, 41].

### Barriers

#### Contextual factors that may deter care-seeking

Seventeen studies showed that newborn care provider training and home visits improved appropriate care-seeking from a health facility by a caregiver at end line compared to baseline or in intervention versus control groups [20–23, 26, 27, 29, 35, 36, 38–41, 45, 49, 50]. Of the studies reporting poor or no difference in care-seeking behaviour, none incorporated consideration of potential barriers to care-seeking within their training and interventions. In one study in India, attribution of newborn illness to “evil eye” or “witchcraft” deterred care-seeking by a caregiver [21]. Conversely, the studies reporting improved care-seeking included innovative methods for increasing community acceptance of -newborn care provision and compliance with provider suggestions such as eliciting perceptions of newborn illness in the community through meetings with community leaders [20], a formal referral card system [26], facilitating transport [26, 29] and a weekly radio program that broadcasted information about healthy postnatal behaviours, newborn danger signs and seeking professional care [27].

Perceptions of gender roles are also an important consideration in community-based newborn care. One study in eastern Uganda highlighted that engaging husbands to support their wives in newborn care was challenging due to a perception that caregiver information on newborn care was designed for women [44]. However, community dialogue meetings led to increasing involvement of men in supporting their wives including saving funds for newborn care and emergencies [44]. Another study in rural Bangladesh included an intervention that specifically targeted husbands and aimed to increase their involvement in newborn care. CBHWs conducted meetings during which they discussed the roles and responsibilities of men in newborn care with the goal of destigmatizing men’s involvement in newborn care [54]. This study demonstrated a significant increase in awareness of danger signs during pregnancy, childbirth and following childbirth among women and their husbands [54].

### Components with unclear benefit

#### Qualifications and experience prior to initiating newborn care training

Newborn care providers’ level of education and competencies prior to initiating training varied widely among studies. Years of school education ranged from at least primary education to a minimum requirement of grade 12 education. In most studies, the minimum required education was at the primary level, with some requiring additional years at the secondary level. Most studies did not mention years of field experience for given CBHW cadres. In one study, CBSV experience ranged from eight months to 25 years with a mean of seven years [34]. Another study stated that 96% of the CHWs had worked for more than five years with few (3.7%) having formal employment [44]. No particular descriptive association emerged between CBHW cadre education and key outcomes. One study in which 90% of CBSVs had at least primary education showed that CBSV assessments and referral decisions for sick newborns achieved excellent agreement with supervisors, suggesting that accurate newborn care provider identification of sick newborns is possible with providers who have at least primary education [20].

#### Newborn care provider incentives or remuneration

Incentives and remuneration for newborn care providers varied widely. Some studies specifically stated that no monetary incentives were offered while others included both monetary and non-monetary incentives. Examples of non-monetary incentives included T-shirts, working materials, briefcases and certificates following training. No clear descriptive association between incentives and key outcomes emerged. One study found through qualitative interviews that incentives were important motivators for CHWs and helped to minimize the dropout rate (3.6%) [44]. At the same time, another study that specified no cash incentives demonstrated high agreement between FCHV identification of sick newborns compared to the more highly trained facility-based CHWs [37].

## Discussion

This review identified 35 articles detailing 28 studies that evaluated CBHW newborn care providers’ ability to recognize sick newborns and improve care-seeking for sick newborns from a health facility in LMICs. Most studies reported that newborn care training and provision can be effective in improving identification of sick newborns and care-seeking from a health facility in diverse LMIC settings where resources are limited and rates of neonatal mortality are high. Key interrelated outcomes that should be measured in an effective newborn care training program include, but are not limited to, high newborn care provider and caregiver knowledge of newborn danger signs, correct identification of sick newborns by providers and caregivers and appropriate care-seeking either through caregiver referral compliance or caregivers themselves seeking care for their newborns based on their knowledge and ability to correctly identify newborn danger signs. Although these outcomes have been identified as important for effective newborn care programs, there is limited understanding regarding the associations between these outcomes and their relative impact on reducing neonatal morbidity and mortality. For example, there is a need to further investigate whether caregiver knowledge of newborn danger signs is associated with improved care seeking. Important components to consider when designing and implementing a newborn care training program to achieve key outcomes were extracted. Facilitators included sufficient duration of training, refresher training and retraining, supervision and on-the-job training and community engagement. Barriers included context-specific understanding of newborn illness and gender roles that may deter care-seeking. Components with unclear benefit included qualifications and experience prior to newborn care training and incentives and remuneration. The studies in this review evaluated key outcomes individually; however, a next step would be to investigate how the components correlate to achieve the desired outcome of improved newborn health.

Duration of training varied widely but less than 11 days of training generally led to poorer outcomes compared to training in the range of weeks to months. The potential benefit of training duration longer than 11 days is important to consider given current WHO recommendations of three to six days for CHW training courses in newborn care [55]. Sufficient duration of training is needed to ensure that newborn care providers are confident in their abilities and that communities, in turn, trust and accept the services of newborn care providers.

Although the ability of newborn care providers to identify danger signs in newborns was generally positive, one study showed a significant decline in provider knowledge of newborn danger signs at one year post-training [44] and another cross-sectional study that did not specify the exact timing of evaluation post-training showed intermediate agreement between ASHAs and postgraduate female investigators regarding identification of sick newborns [31]. This illustrates the importance of routine and periodic feedback and refresher training of newborn care providers in order to maintain their skills in newborn assessment. Evidence of decline in provider knowledge of newborn danger signs at one-year post-training [44] suggests that refresher training should potentially occur earlier than one year. High knowledge retention was demonstrated with monthly retraining by supervisors [42], however, this frequency of retraining may not be practically achievable outside of research settings given limited resources. The optimal timing of refresher training should be further studied.

Similar to refresher training, supervision and on-the-job training were also shown to be essential components of newborn care training programs to reinforce skills and ensure and maintain quality newborn care. Supervision strategies ranged from direct observation to large CBHW group meetings to troubleshoot challenges. Further research is needed on the effects of various supervision strategies on CBHW performance.

Three studies showed no difference between baseline and end line or between intervention and control groups regarding care-seeking from a health facility by a caregiver for a sick newborn. Context-specific perceptions attributing newborn illness to ‘evil eye’ or ‘witchcraft’ deterred caregivers from seeking care from a health facility and perceptions that newborn care information was designed for women deterred the involvement of men in supporting their wives in newborn care. Therefore, more emphasis on developing skills for behaviour change communication and counselling to educate regarding context-specific perceptions of newborn illness and gender roles that may deter care-seeking from a health facility is critical. Beyond simply educating newborn care providers and caregivers about newborn danger signs, community leaders and members need to be actively involved in designing training and interventions in order for the intervention to be trusted, well-received and effective in a given country’s specific socio-cultural context.

Related to culturally-sensitive care, another critical CBHW training component is respectful and dignified care. None of the included studies specified details related to the incorporation of respectful care into training programs. However, given the increasing evidence of disrespect, abuse, mistreatment and stigmatization of newborns [56], future CBHW newborn care training program development should ensure that respectful newborn care provision is taught, evaluated and practiced.

One included study evaluated TBA performance in identifying newborns with danger signs [27]. It is important to acknowledge that although TBAs met our definition of a CBHW, ongoing controversy exists regarding their role in maternal and newborn care including reports of inappropriate and outdated newborn care practices [57]. At the same time, TBAs have also been shown to have a positive role in bridging the relationships between the community and local health facilities [57, 58]. Future newborn care training program development must ensure careful consideration of re-education for TBAs and other CBHWs while acknowledging their potentially important role in health facility referrals in specific cultural contexts and settings.

The importance of prior education and experience and of the presence of incentives and remuneration in improving key outcomes is likely context-specific. Training interventions, and those designing and implementing them, should account for factors that would affect a newborn care provider’s clinical abilities such as educational background, previous training and medical experience, status in the community and remuneration. For example, an ASHA in India is the first point of contact for most communities for primary care and maternal and child health issues and needs to fulfil the educational criteria of being literate with “preference in selection to those who are qualified up to 10 standard … this may be relaxed only if no suitable person with this qualification is available” [59]. The ASHA is not entitled to a salary, but rather receives performance-based incentives depending on the support provided. The potentially diverse education, qualifications and clinical experience of newborn care providers both within and between countries should be considered when determining the duration, content and follow-up in the provision of newborn care training. A broad or heterogeneous definition of what constitutes a CHW or other newborn care provider type may affect planning of interventions particularly with regard to duration and extent of training in a given country’s specific context.

Beyond improving provider and caregiver knowledge of newborn danger signs, identification of sick newborns and appropriate care-seeking through a newborn care training program, it is also important to ensure that transport to a health facility is quickly accessible once the decision to seek care has been made. Studies in LMICs have shown that transport-related delays are a significant contributor to neonatal deaths [8, 9]. In several studies included in this scoping review, newborn care providers facilitated transport to health facilities [20, 29, 30] or a community emergency transport system was established [26, 32, 33]. Functioning referral systems from community to facility are necessary but not sufficient to ensure improved neonatal outcomes. While an effective referral system involving identification of sick newborns and appropriate care-seeking is critical to reduce neonatal mortality, it must be considered as part of a broader continuum of newborn care that includes other important interventions such as breastfeeding support, kangaroo mother care, chlorhexidine umbilical cord cleansing, antibiotic treatment for infections, and full supportive facility care [60]. These interventions should also be taught and knowledge tested in training programs. In addition to functioning referral systems, other critical interconnected system requirements are needed to improve the quality of newborn care including evidence-based practices, actionable information systems, effective communication with patients and families, respect and preservation of dignity, emotional support, a competent and motivated workforce and availability of essential physical resources [61, 62]. As increasing investments are directed at improving inpatient care of small and sick newborns [63], robust training of CBHWs to improve community-based identification of sick newborns and care-seeking from a health facility is needed to ensure appropriate and timely assessment of sick newborns requiring inpatient care.

This scoping review narratively described key components to be considered in designing an effective CBHW newborn care training program to improve identification of sick newborns and appropriate care-seeking. These components align with recommendations from the WHO 2018 guideline on health policy and system support to optimize community health worker programs which include supportive supervision, remuneration and community engagement [64]. Future research in CBHW newborn care training should directly assess the relative impact of various durations of training, timing of refresher training, combinations of supportive supervision strategies, community engagement strategies, mitigation of local perceptions of illness and gender roles that may deter care-seeking, pre-training qualifications and incentives and remuneration on the key outcomes to improve CBHW identification of sick newborns and care-seeking from a health facility.

### Limitations

This review had several limitations. First, as is the case with scoping review methodology, we did not assess the quality of selected studies. The overall high proportion of improved outcomes could be attributed to publication bias. However, this review provides an overview of the available evidence for the effectiveness of newborn care providers in the identification and referral of sick newborns upon which further interventions and strategies can be evaluated and implemented at scale. Second, the included studies only represented 11 countries across three subcontinents and many LMICs were not represented which limits the generalizability of our findings. Future development and implementation of newborn care provider training should be adapted to local cultural, socio-economic and political factors. Third, the time of publication of included articles varied widely with the earliest article published in 2005. Since that time, notable advancements have been made in the evidence base for newborn interventions. These include increased coverage of facility-based deliveries and deliveries attended by skilled health personnel [4], chlorhexidine umbilical cord cleansing, additional evidence supporting known beneficial interventions such as kangaroo mother care and antenatal steroids, outpatient injectable antibiotics for neonatal infections and innovation of equipment and training methods for neonatal resuscitation [3]. Our findings must be considered in the context of these advancements in newborn care. Although such innovations have likely reduced the proportion of sick neonates in community settings, community-based identification of sick newborns and care-seeking from a health facility remains an important priority to continue to reduce neonatal mortality. Finally, no authors of this review were from LMICs. Future research and training program development based on these findings should aim to include LMIC collaborators who could provide important national or local contextual insight.

## Conclusion

Training newborn care providers to successfully identify sick newborns and improve care-seeking behaviour is an important contributor to the global goals of decreasing neonatal mortality and ending preventable deaths of newborns as outlined in the SDG3.2. High newborn care provider and caregiver knowledge of newborn danger signs, correct identification of sick newborns by providers and caregivers and appropriate care-seeking from a health facility are important outcomes to be measured when designing a newborn care training program to reduce neonatal morbidity and mortality. Facilitators to achieve these key outcomes include sufficient duration of training, regular refresher training, supervision and on-the-job training and community engagement. Barriers include local perceptions of newborn illness and gender roles that may deter care-seeking from a health facility. Components with unclear benefit include qualifications prior to training and incentives and remuneration. Training curriculum development must be culturally-sensitive, respectful and grounded within specific local contexts. Our findings can inform the design of future CBHW newborn care training programs in LMICs. Further research is needed to determine the relative impact of the facilitators, barriers and components with unclear benefit on key outcomes and to investigate the associations between key outcomes and their relative impact on reducing neonatal morbidity and mortality.

## Supplementary Information

**Additional file 1.** 1 PRISMA-ScR checklist. PRISMA-ScR checklist.

**Additional file 2.** Full electronic search strategy for Medline (Ovid). Full electronic search strategy for Medline (Ovid).

**Additional file 3.** Characteristics of studies on postnatal care providers in low-and middle-income countries (expanded). Expanded results table.

## Data Availability

The datasets used and/or analysed during the current study are available from the corresponding author on reasonable request.

## Abbreviations

ANM: Auxiliary nurse midwife
ASHA: Accredited Social Health Activist
CBHW: Community-based health worker
CBSV: Community-based surveillance volunteer
CHW: Community health worker
CV: Community volunteer
FCHV: Female community health volunteer
HEW: Health extension worker
LHW: Lady health worker
LMIC: Low- and middle-income country
MDG: Millennium Development Goal
PMTCT: Prevention of mother-to-child transmission
SDG: Sustainable Development Goal
TBA: Traditional birth attendant
VHW: Village health worker
WHO: World Health Organization
